# Characteristics, Compression, and Buffering Performance of Pomelo-Like Hierarchical Capsules Containing Shear Thickening Fluid

**DOI:** 10.3390/polym11071138

**Published:** 2019-07-03

**Authors:** Ting-Ting Li, Junli Huo, Xing Liu, Hongyang Wang, Bing-Chiuan Shiu, Ching-Wen Lou, Jia-Horng Lin

**Affiliations:** 1 Innovation Platform of Intelligent and Energy-Saving Textiles, School of Textile Science and Engineering, Tianjin Polytechnic University, Tianjin 300387, China; 2 Fujian Key Laboratory of Novel Functional Fibers and Materials, Minjiang University, Fuzhou 350108, China; 3 Department of Chemical Engineering and Materials, Ocean College, Minjiang University, Fuzhou 350108, China; 4 Department of Bioinformatics and Medical Engineering, Asia University, Taichung 41354, Taiwan; 5 Department of Medical Research, China Medical University Hospital, China Medical University, Taichung 40402, Taiwan; 6 College of Textile and Clothing, Qingdao University, Shandong 266071, China; 7 Laboratory of Fiber Application and Manufacturing, Department of Fiber and Composite Materials, Feng Chia University, Taichung 40724, Taiwan; 8 Department of Fashion Design, Asia University, Taichung 41354, Taiwan; 9 School of Chinese Medicine, China Medical University, Taichung 40402, Taiwan

**Keywords:** pomelo-like capsules, hierarchical structure, shear thickening fluid, size regulation, compression response

## Abstract

In this study, a double-walled and pomelo-like hierarchical shear thickening fluid (STF) is successfully encapsulated using the simple and environment-friendly calcium alginate encapsulation technique by instilling STF into sodium alginate (SA) and crosslinking by calcium chloride solution. The encapsulated STF has a pomelo-like structure with a shell thickness of 2.9 μm and core pores with a size of 21.43 μm. The effect of the size of STF capsules (2.10, 1.89, 1.86, 1.83, 1.73, and 1.63 mm) is explored in terms of thermal stability, swelling capacity, mechanical property, and release performance. The buffering performance of different sizes of STF-containing capsules is also investigated. The pomelo-like STF capsules can withstand a processing temperature of 250 °C. With a decrease in particle size, the compression strain energy slowly increases first and then rapidly enhances. The kinetic release of pomelo-like STF capsules conforms to Fickian diffusion. STF-containing capsules with a diameter of 1.83 mm present the greatest thermal stability, the highest STF amount, the maximum swelling coefficient, and the fastest kinetic diffusion. STF-containing capsules also have an improved buffering performance in PU foam. This capsule has the best comprehensive performance and can adapt to diversified applications, such as personnel armor and other protective sports equipment.

## 1. Introduction

An increased demand for the quality of life and the increasing popularity of outdoor activities have led us to require outfits that offer functionalities other than comfort and beauty. More people additionally want their clothes to be impact resistant, especially extreme athletes and the elderly. Consequently, people and equipment should be protected from possible danger or risk. Considerable studies on how to improve the impact resistance of materials have been performed. Preliminary studies on natural objects have revealed that reverse engineering can synthesize superior impact-resistant biomimetic composites with hierarchical multi-scaled structures similar to nacre. In addition to impact resistance, flexibility is required for composites to protect users and facilities [1].

Shear thickening fluid (STF) is a non-Newtonian fluid that becomes viscous when the shear stress rate increases. Its viscosity reaches the maximum at a critical shear rate [2,3,4]. STF is commonly used in protective areas because of high viscosity, multiple components, and shear thickening effect [5,6]. Fu et al. added STF to carbon fibers to attain a combined surface with greater shock resistance and energy absorption [7]. Majumdar et al. proposed STF-impregnated woven aramid fabrics to make flexible body armor, which has greater stab resistance properties and strengths [8]. In many studies, STF is added to foam materials to improve their buffering resistance property [9,10,11,12]. Nevertheless, impregnating a matrix with STF is difficult, and removing STF from a substance is easy, resulting in a poor stability. Products made of STF/substance materials are not comfortable and flexible for users. Furthermore, silica (SiO_2_) particles in STF are easily removed from an impregnated substance, which is harmful to human health. STF encapsulation, an effective and simple method, can improve the comfort and prolong the life span of STF impregnated with a substance material.

The encapsulation of STF is rarely studied. Zhang et al. successfully encapsulated STF and yielded double-layered capsules via interfacial polymerization, through which the cortex is polymerized from polyethyleneimine (PEI), diisocyanate prepolymer, and toluene [13]. They used the two-step aggregation method, thereby increasing the operability of STF [14]. However, this method is complex and costly to operate. The selection of a cortex limits the application range of STF capsules. The orifice coagulation bath method has been applied [15,16]. In our previous study, a shear thickener is successfully encapsulated by using the orifice coagulation bath method [17]. As a common and simple encapsulation method, orifice coagulation method has superior characteristics, such as simple device, easy operation, low cost, and uniform particle size. In this method, polymers from a nozzle are formed into capsules with sizes ranging from 500 μm to 1 mm by crosslinking and curing. In the present study, the selected cortex was calcium alginate (sodium alginate) cross-linked by calcium chloride. Calcium alginate, as a biocompatible and non-immunogenic polymer, is degradable, cheap, and easily industrialized [18,19]. STF capsules which have a pomelo-like hierarchical structure, were prepared via the orifice coagulation method. The effect of the size of STF capsules is explored in terms of thermal stability, swelling capacity, mechanical property, release performance and buffering performance. The determined optimal capsule size could be used as a reference for conducting further studies.

## 2. Experimental Section

### 2.1. Materials and Methods

PEG 200 has an average molecular weight (MW) of 190–210. Liquid paraffin has a relative density of 0.835–0.855. Anhydrous ethanol, sodium alginate (SA), and calcium chloride have mass fractions of ≥99.7%, 90%, and ≥96%, respectively. SiO_2_ has a size of 500 nm, a mass fraction ≥99.5%, and a MW of 60.08. Span 80 (MW = 428.61) and Span 20 (MW = 346.46) were purchased from Tianjin Sanjiang (Serida) Technology Co., Ltd., Sanjiang, China.

### 2.2. Preparation of STF

PEG 200 (40 mL) was stirred in a beaker at 100 r/min, Afterward, 500 nm SiO_2_ (10 g) and anhydrous ethanol (80 mL) were infused. The mixture was blended with an agitator at 600 r/min for 6 h. processed with ultrasonic oscillation for 3 h, and dried in an oven for 24 h to remove the air bubbles. STF was obtained after anhydrous ethanol completely evaporated.

### 2.3. Preparation of STF Capsules

**Figure 1:**
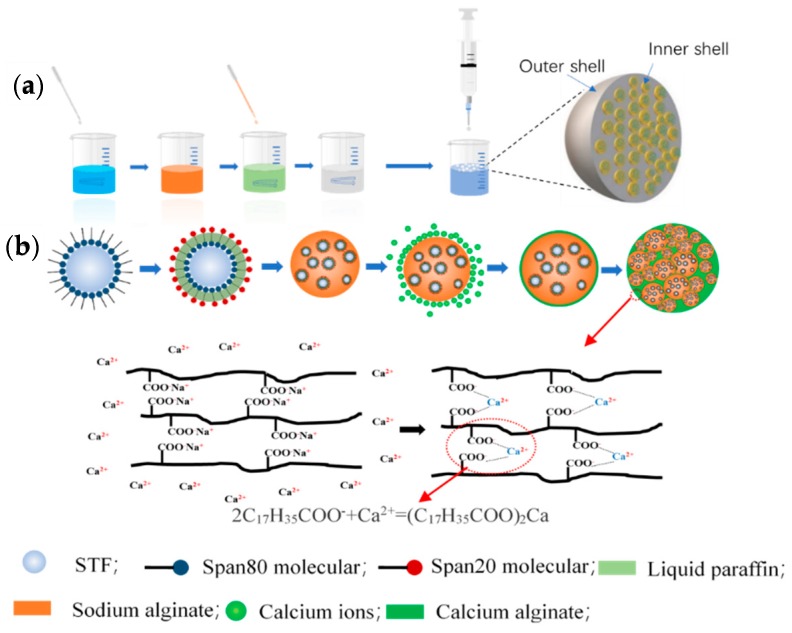
(**a**) Preparation process of STF capsules and (**b**) the schematic diagram of reaction principle for the shells of capsules.

**Figure A1:**
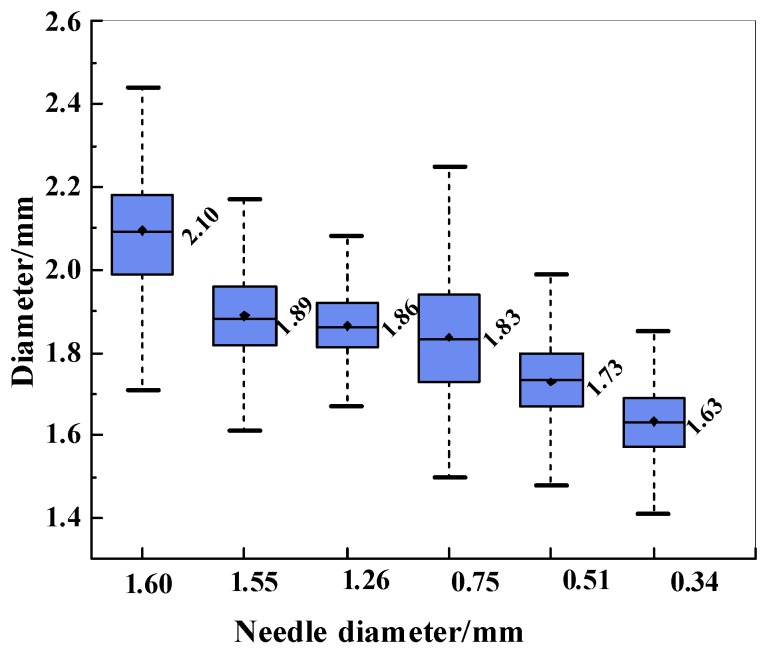
Capsule size distribution as related to the needle diameter.

Figure 1a shows the process of producing STF capsules. Hydrophobic surfactant (Span 80) was added to liquid paraffin and fully mixed. Next, STF was dropped into liquid paraffin, magnetically stirred at 1000 r/min at 25 °C for 30 min. The final product was a water-in-oil (W/O) emulsion) called colostrum, A hydrophilic surfactant (Span 20) was added to a sodium alginate solution, fully mixed, and slowly added with colostrum. The mixture was mixed with a magnetic stirrer at a speed of 100 r/min at 25 °C for another 20 min, thereby forming a water-in-oil-in-water (W/O/W) multiple emulsion [20,21]. W/O/W multiple emulsion was sucked into a 10 mL syringe with a specified orifice diameter of 1.55 mm. The syringe was then fixed to a microinjection pump, which operated at a driving speed of 10 μL/min, pushing multiple emulsion into a prepared calcium chloride solution to form spherical droplets. Subsequently, the crosslinking between sodium alginate and calcium chloride provides the STF capsule with a solid surface [22]. The collected capsules were rinsed thrice with deionized water and dried on a water/oil absorbent paper at room temperature. The calcium-alginate shells of STF capsules were formed as a result of the cross-linking of sodium alginate and calcium chloride. Six kinds of particle size capsules (2.10, 1.89, 1.86, 1.83, 1.73, and 1.63 mm) were prepared in this study, and the particle size distribution is shown in Figure A1.

In Figure 1b, sodium alginate containing STF and liquid paraffin was dropped into a calcium chloride solution. Large Ca^2+^ concentrations from calcium chloride solution replaced Na+ from sodium alginate and then cross-linked with –COO– to form calcium alginate. STF capsules have a liquid STF core and a hierarchical pomelo-like shell structure because W/O/W drops were sealed in the capsules during the crosslinking and curing of shells. Calcium alginate does not dissolve in water and serves as capsule shells [20,22,23].

### 2.4. Measurements and Characterizations

A Malvern rotational rheometer was used to measure the rheological properties of STF. Flat plates (pp40) with a diameter of 4 cm were fitted 3 mm apart. The rheological rate range was 0.1–1000 r/min at 25 °C. The functional groups of STF, W/O/W multiple emulsion, and shells are analyzed using a Fourier transform infrared (FT-IR) spectrometer (NICOLET iS10, Thermo Fisher Scientific, Waltham, MA, USA). The wavelength range and resolution were 400–4000 cm^−1^ and 4 cm^−1^, respectively. The morphological characteristics of surface and interior of the capsules were observed using a scanning electron microscope (SEM, TM3030, HITACHI, Tokyo, Japan). The diameter of the cavity inside the capsule was measured with Image-Pro Plus (6.0, Rockville, Maryland, USA), and the number of cavities counted was 100. The thermal stability of the capsules is measured using a thermogravimetric analyzer (TG 209F3, NETZSCH, Bavaria, Germany) with nitrogen as the shielding gas. The samples (5–10 mg) were heated from room temperature to 600 °C at a rate of 10 °C/min. The particle size of the capsule was observed using a stereomicroscope (Nikon SMZ-10A, Taiwan, China).

The expansion degree of capsules was characterized by diameter expansion coefficient (*DEC*) and weight expansion coefficient (*WEC*). The capsules were immersed in deionized water for 24 h, and their diameter and weight were measured. The results before and after immersion were compared to compute DEC and WEC. The employed equations were as follows [15]:

(1) $$DEC=\frac{D_{s}}{D_{d}},$$

(2) $$WEC=\frac{w_{s}}{w_{d}},$$

where *D_s_* and *D_d_* are the average diameter of swollen and dried microcapsules, respectively, while *W_s_* and *W_d_* are the weight of swollen and dried beads, respectively.

**Figure A2:**
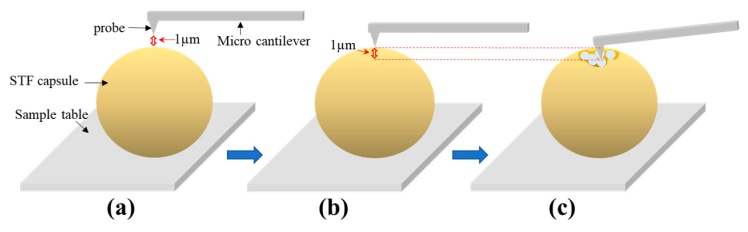
Schematic diagram of atomic force microscope test process, probe (**a**) before puncture, (**b**) at the time of puncture, and (**c**) after puncture.

The bearing load of STF capsules is used to characterize their application to the environment. The inner and outer shells of the STF capsules were measured using an atomic force microscope (AFM, Agilent 5500, AGILENT Company, Palo Alto, CA, USA). The elastic coefficient of microcantilever of the probe had a constant force of 40 N/m, and the descending height of the probe was set to be ±1 μm as displayed in Figure A2. The static compression strength of capsule was measured at a rate of 5 mm/min by using a universal testing machine (HT-2402, Hong Ta Instrument, Taiwan, China) as specified in ASTM D 3574-17.

**Figure A3:**
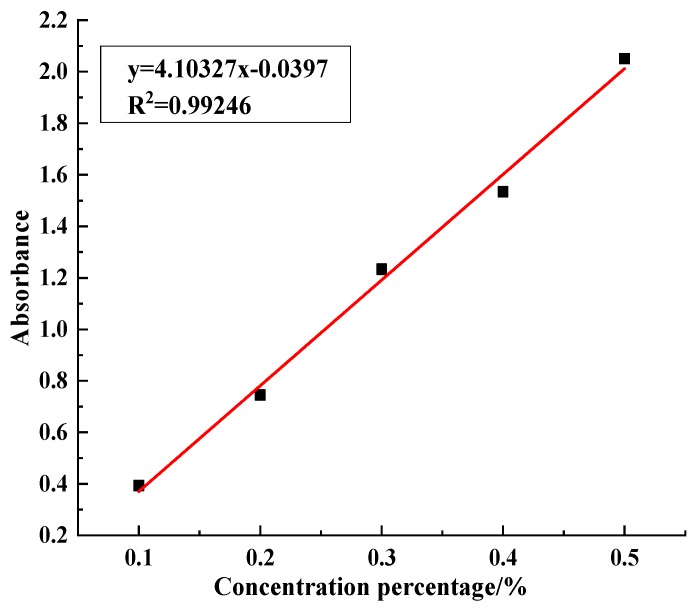
Standard curve of STF.

The slow-release and kinetics of the capsules were measured using a UV–VIS spectrophotometer (UH4150, Hitachi, Japan). The wavelength range was 200–800 nm. The characteristic wavelength of STF was observed at 203.5 nm, which was consistent with that described in other references [24,25]. Different concentrations of STF solutions (0.1%, 0.2%, 0.3%, 0.4%, and 0.5%) were formulated to obtain the standard curves of absorbance at 203.5 nm (Table A1 and Figure A3). The correlation of absorbance (Y) and STF concentration (X) was displayed as Y = 4.10327X − 0.0397 (R^2^ = 0.98).

In the slow-release performance test, the capsules were soaked in deionized water and sealed for storage for 1, 2, 4, 8, 12, 24, and 72 h. The soaking liquids are then removed and their absorbance was measured. The absorbance with its standard curve could be computed to determine the concentration of the soaking solution, Afterward, the amount of the released STF were compared and observed in relation to different capsule sizes. A Korsmeyer–Peppas model was used to interpret the release kinetics type of STF as follows [26,27]:

M_t_/M_∞_ = K t ^n^ (3)

where M_t_ is the amount of STF at a specified time (t), M_∞_ is the total amount of STF in gel beads, K is the release kinetic constants, and n is the release exponent.

After encapsulation was completed, STF was added to the PU foam for a drop-weight impact tester. The foam was prepared in accordance with our previous study [28]. The drop-weight impact tester was self-made by the Xinzhi Electronic Automation Company (Taichung, Taiwan). Different sized STF capsules were filled into the PU foam to evaluate their buffering performance. The impactor freely dropped from a constant height and impacted on the surface of the resultant STF capsule-filled foam. The impactor head was circular and had a diameter of 32 mm. The impact energy was 20 J. The sample had a size of 100 mm × 100 mm.

## 3. Results and Discussion

### 3.1. Characterization of STF Capsules

**Figure 2:**
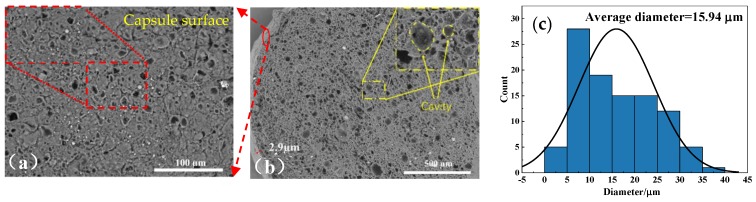

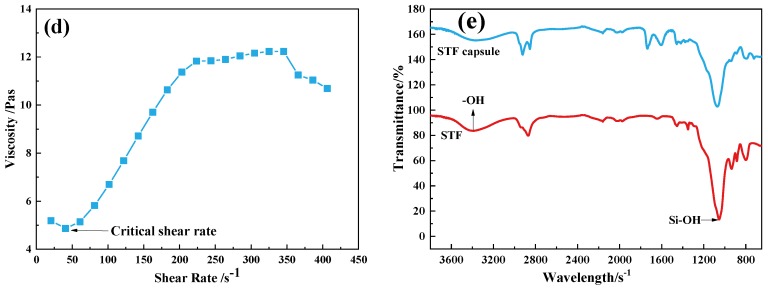
SEM observations of (**a**) capsule shell and (**b**) cross section of STF capsules; (**c**) cavity diameter distribution in the inner of STF capsules; (**d**) rheological curve of STF; and (**e**) FTIR spectrum curves of STF and STF capsules.

Figure 2a,b show the SEM images of surface and cross-section of STF capsules, respectively. It is found that STF capsules have a structure resembling the hierarchical pomelo peel. The surface of the capsule is rough, indicating resembles the pomelo peel, which contains a compact shell layer covering the internal micro-pores filled with liquid [29]. The outer dense shell has a thickness of 2.9 μm. Figure 2b shows that the surface is composed of numerous cured sodium alginate droplets that seal liquid paraffin and STF. The interior of capsules was composed of many tubular cavities with a structure comparable with that of pomelo peels [30]. During STF capsule preparation, calcium chloride solution on the surface of the capsule and infiltrated into the capsule sphere is cross-linked with the sodium alginate on the outermost layer of W/O/W, forming water-insoluble calcium alginate, which has a dense layer on the surface of the capsule and the cavity wall inside the capsule. Calcium chloride and the outermost layer of sodium alginate in W/O/W emulsion is cross-linked because the capsule is composed of a large number of closely arranged W/O/W complex emulsion droplets, forming an insoluble calcium alginate surface wrapped with W/O emulsion microspheres. The interior of STF capsules is composed of many tubular cavities by the cross-section observation because the existence of calcium alginate makes the microspheres adhere to one another. The average diameter of the cavity is 15.94 μm as shown in Figure 2c.

Sealing STF in the capsules aims to maximize the advantage of its shear thickening feature as shown in Figure 2d. The viscosity of STF decreases as the shear rate increases; when viscosity is the lowest, it upsurges rapidly. This process is called shear thickening [7,31]. As a result, when the capsule is subjected to an external force, STF rapidly increases and absorbs some impact energy to attain the buffering efficacy.

The FTIR curves of STF and STF capsule were obtained to verify whether STF is encapsulated (Figure 2e). The FTIR curve of the STF capsules had Si-OH characteristic peaks [32], and this observation was consistent with a pure STF curve, indicating that STF was successfully filled. The peak at 3328 cm^−1^ corresponds to -OH anti-scaling vibration peak in constitution water. The presence of peaks at 1062 cm^−1^ and 932 cm^−1^ were due to Si-O-Si anti-scaling vibration and Si-OH bending vibration, respectively. Si-O symmetric stretching vibration peak occurs at 480 and 814 cm^−1^ [33]. The four characteristic peaks showed the existence of SiO_2_ in STF capsules. STF had similar FTIR spectra, indicating that the STF was successfully sealed in the capsules, where all the required components were present.

### 3.2. Effect of Capsule Size on Thermal Stability

**Figure 3:**
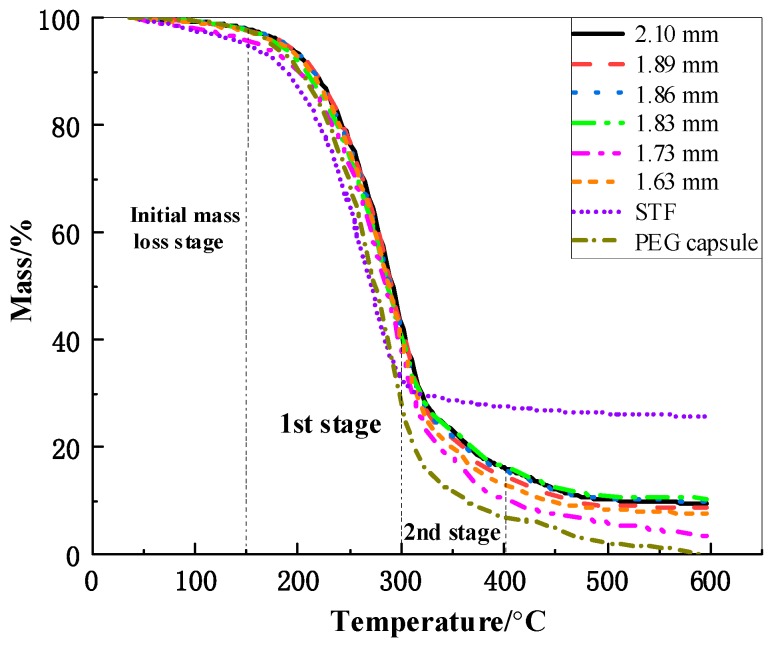
TG curves of different sizes of STF capsules.

The thermal stability of STF capsules can reflect the strength, stability in the application process, and application temperature of capsules [34,35]. With TG analysis, the thermal stability of the capsules is characterized effectively [36]. Figure 3 shows the TG behaviors of the capsules in relation to their sizes. Initial weightlessness temperature (TInitial), first- and second-stage maximum mass loss (1st Tmax, 2nd Tmax) and residual mass are displayed in Table 1. Other than STF, similar shape and decomposition appears in the TG curves at two stages: 200 °C–300 °C (first stage) and 300 °C–400 °C (second stage). The initial mass loss of the STF capsules began at 40 °C–200 °C, which is ascribed to the evaporation of water and solvent from capsules [37]. In the first-stage loss, the mass loss is ascribed to the dissolution of SA segments, the removal of SA lateral groups, the random depolymerization of SA, and the decomposition of liquid paraffin, whose decomposition temperature is 234.8 °C. The second- stage decomposition may be attributed to secondary degradation, including the cleavage and pyrolysis of PEG chains at around 300 °C [38].

The initial loss temperature and the first decomposition temperature of the STF capsules were higher than those of pure STF, indicating that SA encapsulation improved the thermal stability of STF. The initial loss temperature and the first decomposition temperature increases by 17.7 °C and 11.8 °C, respectively. The residue represents the amount of SiO_2_. In Table 1, 1.83-mm capsule had a maximum residual mass of 10.38%, indicating the maximum amount of STF in the capsules. In conclusion, the pomelo-like hierarchical capsules containing STF fabricated in this study could resist temperatures below 200 °C.

### 3.3. Effect of Capsule Size on Capsule Strength

**Figure 4:**
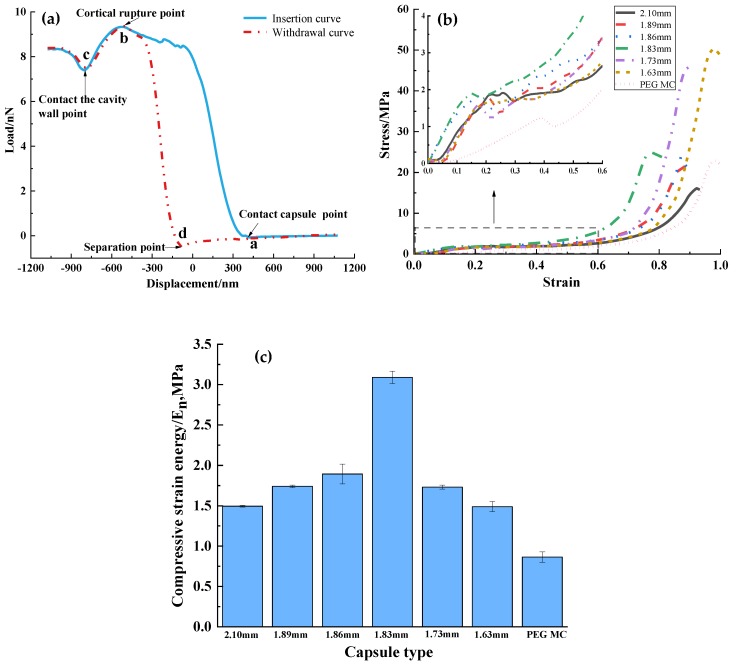
(**a**) Instantaneous force-displacement curves by AFM; (**b**) static compression stress-strain curve, and (**c**) at 60% compression strain, compressive strain energy of a single capsule as related to different capsule sizes.

Figure 4a shows the instantaneous force-displacement curve via AFM, involving the probe being employed forward and backward, i.e., insertion and withdraw movements [39]. When the probe tip came in contact with the STF capsules and reached the contact capsule point (point a), the force gradually increases. As the probe tip pierced into the STF capsules reaching the cortical rupture point (point b) and entered into foam-like pores, the compact surface wall of STF capsules was damaged, and the force began to decline. After the probe tip penetrated the cavity (point c), the force increases gradually again because of the interior of the capsules in the cavity. The withdraw force related to probe tip displacement reflected the properties of the STF capsules after the damage. When point d was reached, the probe tip was separated from the STF capsules. This displacement of the separation point was earlier than that in the insertion curves. This finding may be caused by the adhesive attraction between the probe tip and the capsule [39]. The observed AFM curves also suggested that the capsules were composed of a core-shell structure. The capsule strength differed as a result of the continuous increase in force possibly because of the fracture of small particles on the capsule’s surface and the destruction of the multilayer structure of the capsules.

When the probe moves backward, strength increases as a result of the resistance of the capsule shells. when the probe is removed from the capsules, strength decreases. Figure 4b shows the stress-strain curves of STF capsules and PEG capsules. The strength and toughness of materials can be determined by observing the stress-strain curve [40]. The inset shows that the capsules have stress that is proportional to the strain regardless of capsules size. Stress decreases at a certain strain. Outer shells are solidified into a hard outer shell because the droplets on the capsule surface undergo complete crosslinking with calcium chloride. The outer wall of shells break under a certain pressure, and the stress correspondingly decreases at the spot. As strain continuously increases, the stress enters a platform region, indicating that solid droplets inside the capsules break and STF demonstrates that its shear thickening efficacy achieves balance with stress. Afterward, the droplets inside the capsules are completely broken and compressed into a concrete structure, Afterward, stress rapidly increases. In general, stress drastically decreases as a result of the presence of completely ruptured capsules.

The slope of the stress–strain curve represents the modulus of elasticity. The greater the slope is, the greater the capsule stiffness at initial compression will be. In the magnified image in Figure 4b, when the capsules are being initially compressed, they show the slope from the highest to the lowest at corresponding capsule sizes of 1.83, 1.86, and 1.63 mm. The 1.83 mm capsules are confirmed to have the highest strength and stiffness, indicating that the capsules exhibit the highest degree of the outer shell of crosslinking. Hence, the resulting calcium alginate dense shell is mechanically strengthened. When the capsules are completely damaged, the maximum stress decreases when the capsule size increases.

PEG capsules (2.10 mm) were prepared to compare with STF capsules in terms of the mechanical properties. The test results show that PEG capsules had the lowest stress, which verified that STF capsules were effective in buffering efficacy. Figure 4c shows the histogram of strain energy base on the stress strain curves of STF and PEG capsules. The strain energy of the capsules with a particle size larger than 1.83 mm increased as the particle size increased. By contrast, the strain energy of the capsules with a particle size smaller than 1.83 mm decreased as the particle size decreased. A small capsule size indicated that the shell had a higher crosslinking degree and, thus, a higher stiffness. Nevertheless, highly stiff shells fail to reflect the powerful buffering effect of STF when an external impact force is applied to small capsules. The buffering efficacy of capsules is exemplified by 60% of the initial compressive stress-strain values. In particular, 1.83-mm capsules had a maximum stress strain energy, which was 16.7 times higher than that of the STF capsule studied by Zhang H et al. [13]. The STF capsule in this study had a Pomelo-like hierarchical porous structure which increases its compression energy. The 1.83-mm capsule contained the greatest amount of STF, which contributed to cushioning efficacy, because of its excellent shear thickening performance.

**Figure 5:**
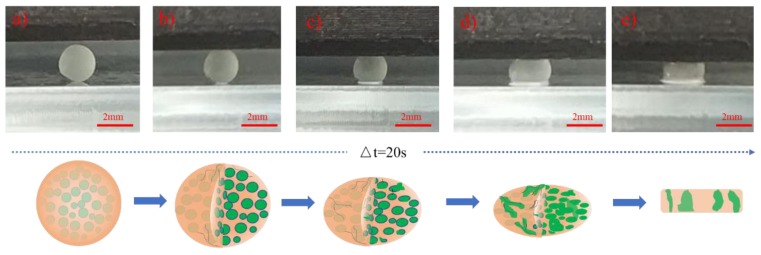
Schematic diagrams of compression deformation process of single capsule during Δt = 20 s.

Figure 5 illustrates the action mechanism of interior pores and STF when the capsules are under a pressure. The capsules are also compressed into a round drum as a result of a deformation after they are compressed for 20 s. In response to an increase in compression displacement, the ongoing deformation of capsules extrudes more liquid from the capsules. When being totally compressed into cuboids, the capsules lose the contained liquid. The diagrams beneath Figure 5 show the internal mechanism when capsules are compressed.

### 3.4. Effect of Capsule Size on Cavity Distribution

**Figure 6:**
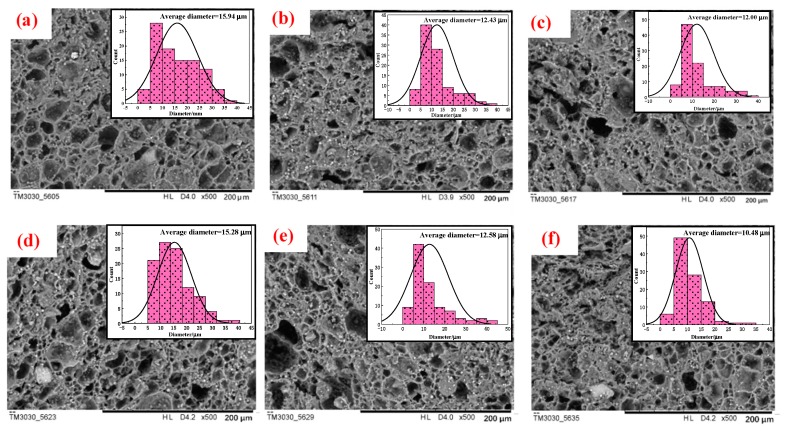
SEM images and cavity distribution of capsules at different sizes (500×), (**a**) 2.10 mm; (**b**) 1.89 mm; (**c**) 1.86 mm; (**d**) 1.83 mm; (**e**) 1.73 mm; and (**f**) 1.63 mm; respectively.

Figure 6 shows the SEM images of the cross-sections of interior capsules at different sizes, suggesting that the size and number of the interior droplets vary. The average diameters of the capsule cells with increasing capsule sizes are 15.94, 12.43, 12.00, 15.28, 12.58, and 10.48 μm. The swelling and stability of the capsules at different sizes are dependent on the size of capsule cells. Subsequently, a capsule size of 2.1 mm indicates that the capsules are composed of larger droplets that are more evenly distributed. By contrast, the 1.89-mm capsules are composed of smaller droplets that are not evenly distributed. Comparatively, the 1.83-mm capsules demonstrate a sharp increase in stability, which may be described as larger interior cured pores that are evenly distributed and highly concentrated. Thermal stability (TG) analysis shows that the 1.83-mm capsules encapsulate the greatest number of STF. In the capsule soaking process, STF permeates through the capsules, causing the interior cavities to swell. Water molecules penetrate the capsules, thereby increasing the swelling coefficient rapidly.

### 3.5. Slow Release Performance and Diffusion Mechanism

**Figure 7:**
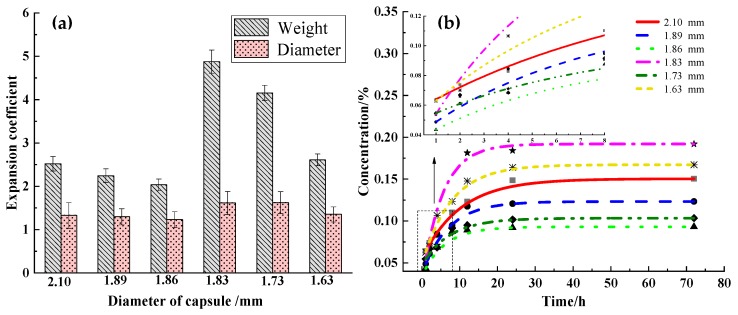
(**a**) Diameter and weight swelling coefficient and (**b**) The simulated absorbance value curves of STF-contained capsules at different sizes as related to the deionized water immersion time being 1 h, 2 h, 4 h, 8 h, 12 h, 24 h, and 72 h.

Figure 7a,b show the swelling test results, about the diameter and weight swelling and the absorbance of STF capsules that are immersed in deionized water for different durations [26]. When the average capsule diameters are 2.1, 1.89, and 1.86 mm, the diameter and weight swelling of the capsules decrease to some degree. When the diameter of the capsule decreases to 1.83 mm, the diameter and weight of the capsules upsurge drastically, but gradually decrease when the capsule diameter exceeds 1.83 mm (Figure 7a). Figure 7b shows that, the absorbance of the capsules from large to small are 0.06392, 0.04868, 0.04341, 0.05376, 0.05464, and 0.06280 for 1 h of immersion. Specifically, the 1.86 and 2.10-mm capsules have the lowest and the highest absorbances, respectively. A long immersion time contributes to a rapid increase in absorbance regardless of capsule size. After 24 h of immersion, the absorbance of the capsules increases more slowly, suggesting that capsules exhibit a stable release performance. With an immersion time of 72 h, the absorbance of the capsules from a large to small size are 0.15044, 0.12334, 0.09313, 0.19202, 0.10341, and 0.16723. In particular, 1.86 and 1.83 mm capsules have the lowest and the highest absorbance, respectively. The 1.83 mm capsules have an absorbance that sharply increases between 8 and 12 h because they seal the maximum STF and have evenly distributed large interior pores.

Table 2 shows the simulated results of the capsules at different sizes by using the Korsmeyer–Peppas model. A greater R^2^ indicates that it fits the description of the release mechanism. At the same time, the release exponent (n) illustrates the release mechanism of STF. As far as a spherical transmission system is concerned, n between 0 and 0.5 confirms that it is Fickian diffusion that follows the Fickian’s law [41]. Moreover, k in the Korsmeyer–Peppas model represents the release kinetic constant. The higher k is, the more efficient the diffusion velocity will be [42]. At R^2^ between 0.771 and 0.902, the 2.10-mm capsules fit this specific release mechanism. n is between 0.140 and 0.289, suggesting that all capsules have a release performance as stated in Fickian diffusion, regardless of capsule size, especially 1.63-mm capsules, whose k corresponds to the most efficient diffusion.

### 3.6. Effects of Capsule Size on Buffering Performance of Capsule-Filled Foam Composites (CPUC)

**Figure 8:**
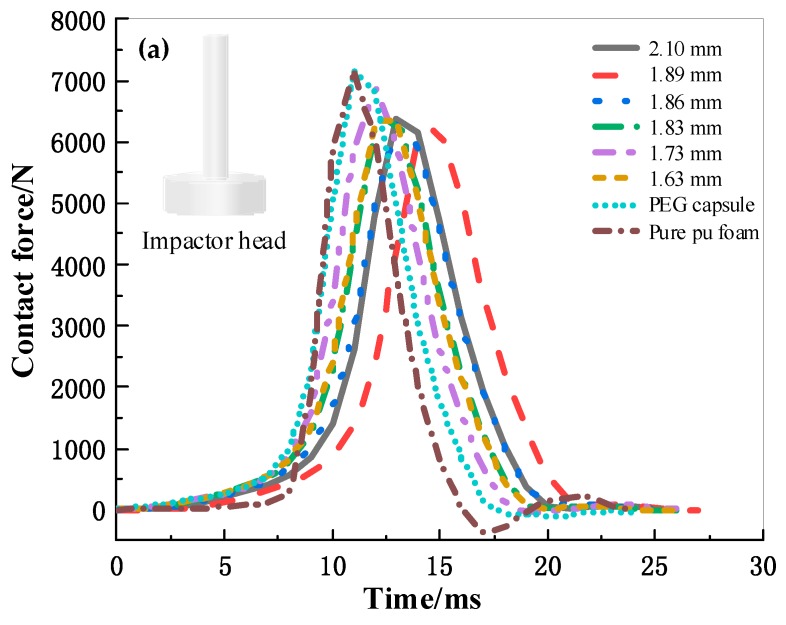

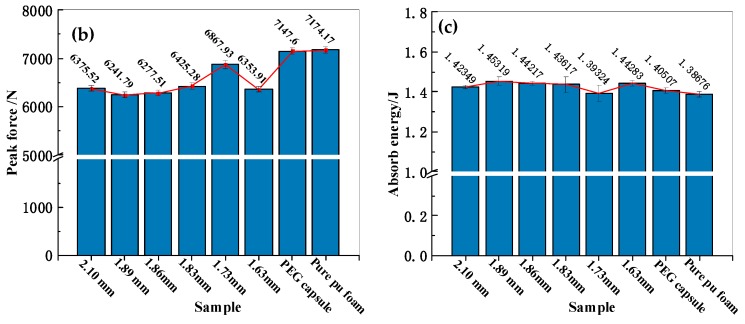
Impact signals at 20 J of CPUC. (**a**) Contact force-time curve of CPUC; (**b**) peak force of CPUC; and (**c**) energy absorption of CPUC.

The STF capsules were added to the PU foam, forming CPUC, to study the buffering performance of STF capsules. The foaming process is similar to our previous study [28]. Figure 8a shows the three stages of the time-contact force curves of different CPUC samples. In stage I, the contact force gradually increases with time, and this procedure is similar to the plateau stage of static compressive curves. In stage II, the contact force rapidly increases with time, corresponding to the densification stage of the static compressive curves of composites. In stage III, the contact force rapidly decreases. Comparatively, PEG capsules and pure PU foam have the shortest plateau region, and their contact force reaches the maximum at 11 ms. The length of the plateau region is followed by 1.73, 2.10, 1.86, 1.83, and 1.63 mm CPUC successively from low to high, corresponding to the maximum contact force happening at 12, 13, 13, 13, 13, and 15 ms. The length of the plateau region expresses the speed of initial deformation of CPUC, indicating that STF capsules prolong the deformation time which is positively correlated with buffering performance and ascribed to the existence of STF and the pomelo-like hierarchical structure of capsules. With small STF capsules, the maximum contact force initially decreases, subsequently increases, and then decreases. The STF capsules with a size of 1.63 mm absorbed the highest impact energy because smaller capsules reinforce the crosslinking density and increase the absorbed impact energy [43]. The maximum contact force of 1.89 mm CPUC is lowered by 12.67% compared with that of the PEG capsule (7.15 kN). CPUC with a size of 1.73 mm has the highest maximum contact force, which is due to the low amount of STF. This observation was consistent with the TG results shown in Figure 3. The tendency of the absorbed energy related to the STF capsule size is opposite to that of the maximum contact force. Comparatively, the pure PU foam and the foam with PEG capsules have the shortest plateau region, and their contact force reaches the maximum at 11 ms. CPUC filled with 1.89 mm STF capsule absorbed the highest impact energy reaching 1.45 J at 3% mass fraction, which is 3.4% higher than that of the PEG capsule and 4.57% higher than that of pure PU foam. This improvement can be further increased by increasing the STF amount and the STF capsules content in the foam composites.

## 4. Conclusions

In this study, sodium alginate is cross-linked with calcium chloride for curing as gel shells that encapsulate STF. The diameter of the syringe needle is adjusted to produce STF capsules with different capsule sizes. The STF capsules are characterized by SEM, rheological properties, and FTIR spectroscopy. The effects of the size of pomelo-like hierarchical STF capsules on thermal stability, swelling capacity, mechanical property, release kinetics, and buffering performance are explored in depth.

SEM confirms that STF capsules have double-walled shells and porous core hierarchical structure, thereby assembling pomelo peel. The size of the STF capsule affects thermal stability, compression energy at 60% strain, slow-release speed, and absorbed impact energy. TG result shows that 1.83 mm capsules outperform other capsules because of the highest amount of STF and the ability to withstand a temperature of 200 °C. This finding corresponds to the tendency of compression resistance and release kinetic, that is, 1.83 mm STF capsules have the highest compression energy and slow-release speed. Comprehensively, the resultant pomelo-like hierarchical STF-containing capsules have superior thermal stability (<200 °C), compression energy, and absorbed impact energy, and STF diffusion conforms to Fickian diffusion. This study provides a preferable prospect for future STF applications. In the following study, we will focus on how to enhance the sealing efficiency of STF and the content of STF capsules to further improve the buffering performance of STF capsules-filled composites.

## Figures and Tables

**Table 1:** TG results of STF capsules with different particle sizes.

Capsule Size/mm	TInitial/°C	1st Tmax /°C	2nd Tmax /°C	Residual Mass Percentage/%
2.10	219.7	296.8	367.3	9.54
1.89	235.4	296.3	364.7	8.67
1.86	224.7	295.6	367.3	9.85
1.83	219.1	294.1	367.2	10.38
1.73	224.3	297.0	362.4	3.28
1.63	232.5	295.8	362.3	7.46
STF	214.8	285.2	/	25.77
PEG capsule	223.5	290.1	362.4	0

**Table 2:** Effect of capsule size on STF release kinetics.

Diameter of Capsule/mm	R^2^	Release Exponent (n)	Release Kinetic Constant (k)	Release Mechanism
2.10	0.902	0.202	0.069	Fickian diffusion
1.89	0.825	0.185	0.062	Fickian diffusion
1.86	0.771	0.141	0.056	Fickian diffusion
1.83	0.774	0.289	0.063	Fickian diffusion
1.73	0.848	0.140	0.062	Fickian diffusion
1.63	0.856	0.208	0.076	Fickian diffusion

## Appendix A

Figure A1 is the boxplot of capsule particle size distribution prepared with needles of different diameters. On averagely, the diameter of the capsules is greater than that of the needles because droplets merge to a certain volume when they are dropped. After the capsules are dropped into a calcium chloride solution, the bulk droplets undergo crosslinking and subsequent curing and are transformed into capsules with increasing diameter. The diameters of all the groups are normally distributed regardless of capsule size. The normal distribution of capsule sizes has a relatively narrow transverse span, suggesting that capsule size is relatively concentrated, especially the capsule size of 1.73 mm.

Figure A2 shows a schematic of the relationship between the probe and the capsule position during the testing of the mechanical properties of the capsule by atomic force microscope (AFM). Figure A2a shows that the probe is 1 µm away from the surface of the capsule when the probe begins to descend, and the probe is driven downward by using a microcantilever. Figure A2b illustrates that the tip of the probe is in contact with the surface of the capsule when the probe moves down to the surface of the capsule. A displacement point where the force starts to increase in the force-displacement curve may not be at zero. Figure A2c demonstrates that the probe continues to move down by 1 µm. The tip of the probe penetrates through the capsule cortex, punctures the capsule cortex, and touches the cavity wall inside the capsule.

**Table A1:** Relation between concentration of SiO_2_ and absorbance.

STF concentration/%	0.1	0.2	0.3	0.4	0.5
Absorbance	0.393467	0.7453	1.232833	1.5341	2.0507
